# Outcomes of decompensated chronic liver disease in a UK district general hospital critical care setting

**DOI:** 10.1186/cc14460

**Published:** 2015-03-16

**Authors:** E Ahmadnia, F Manneh, K Raveendran

**Affiliations:** 1 Homerton University Hospital, London, UK; 2 Queen's Hospital, London, UK

## Introduction

Patients with decompensated cirrhosis admitted to the ICU have historically had a very high mortality rate [1]. It has been suggested that improving patient selection can improve ICU outcomes in patients with cirrhosis [2]. The aim of this study was to determine the mortality and evaluate the risk factors that may influence the outcome of this group of patients in a large UK district general hospital with a view to introducing selection criteria for future ICU admission of patients with decompensated liver disease.

## Methods

A retrospective analysis was performed of all adult patients with decompensated chronic liver disease admitted to a general (nontransplant) critical care unit between January 2012 and December 2013. Data were collected regarding demographics, ICU mortality, hospital mortality, aetiology of chronic liver disease, severity scores, acute diagnoses, and organ support requirements.

## Results

Thirty-seven patients were identified, with a median age of 57 years, predominantly male (62%). Seventy-six per cent had alcohol-related cirrhosis. Overall ICU mortality was 29.7% and hospital mortality was 48.6% - these values were higher in the alcoholic group (39.3% and 57.1% respectively). All ICU deaths were in those with alcoholic liver disease. Median scores were: APACHE III 93, SOFA (day 1) 9, Child-Pugh 11, MELD 21. Seventy per cent were treated for sepsis, 22% had a GI bleed, 57% had encephalopathy, 24% had suspected/ confirmed spontaneous bacterial peritonitis, and 70% had an acute kidney injury. Organ support requirements were: 35% respiratory (non-invasive or invasive ventilation), 38% vasoactive agent support, 24% renal replacement therapy (RRT). Alcoholic liver disease patients requiring respiratory or cardiovascular support had an ICU mortality of 64%, and those requiring RRT had a mortality of 75%. Alcoholic liver disease patients requiring combined respiratory, cardiovascular, and RRT support had 100% mortality.

## Conclusion

Those with decompensated chronic liver disease admitted to the ICU have a significant ICU/hospital mortality, which is increased in alcoholic liver disease. Sepsis and AKI were the most common acute diagnoses in this cohort. Alcoholic liver disease patients requiring organ support have a very high mortality, and the outlook for multiorgan failure requiring RRT in this group is dismal.
